# Why We Can Thrive past Seventy‐Five: In Favor of Efforts to Extend the Human Lifespan

**DOI:** 10.1002/hast.5007

**Published:** 2025-06-25

**Authors:** Carolyn B. Ringel, Michael S. Ringel, Arthur L. Caplan

**Keywords:** aging biology research, longevity, bioethics, health span, health care

## Abstract

*About ten years ago, Ezekiel Emanuel wrote an article extolling the benefits of dying at seventy‐five. Since then, longevity and aging interest, research, and funding have exploded. Much of the public is supportive of aging biology research, and books on extending the human lifespan populate bestseller lists. However, the issue remains hotly debated, and many articles published in the lay press spin the research in a negative light. Yet, if we collect these arguments and address each one logically, we see that each implies untenable conclusions. More to the point, there are strong arguments that human health and life have fundamental value and that incremental gains in health and in years of life will benefit us. For both ethical and practical reasons, we should support aging research*.

## Essay

In 2014, Ezekiel (“Zeke”) Emanuel published an oft‐cited essay titled “Why I Hope to Die at 75.”^1^ He argued why one should go gentle into that good night after three‐quarters of a century of life (and just a little before the average life expectancy for a white male in the United States). Emanuel took the position that, after age seventy‐five, a person's cognitive and physical capacities decline in such a way that their remaining years are an unpleasant burden on both the person and those who love him or her. The title was calibrated to be shocking; a little over fifty years ago, the sociologist Ernest Becker asserted that essentially all human anxiety was motivated by a fear of death and that humans spend a lifetime pushing back against the idea that they must one day die.^2^

In the ten years since that essay appeared, as the clock ticks on the now sixty‐seven‐year‐old Emanuel, there has been an explosion in research on aging biology. “Aging biology” refers here to aspects of biology that have been shown to extend lifespan in other animals: things like insulin signaling (which kicked off the field by showing that silencing a single gene in this pathway could nearly double a worm's lifespan), clearance of senescent cells (“zombie‐like” cells that have lost function and, worse, secrete inflammatory signals to the cells around them), and epigenetic reprogramming (the same technology that enables cloning and that has been shown to extend remaining lifespan of aged mice by more than 100 percent). The core premise of this discipline is that the rate of aging can be modulated and that science can thus extend lifespan and—because so many of the diseases that plague humans are consequences of aging—health span, the period free of disease. The book *Outlive: The Science and Art of Longevity*, by Bill Gifford and Peter Attia, is on the *New York Times*’ bestseller list. Conferences are held in the Americas, Europe, and the Middle East to explore the science of healthy aging. The supplement business is booming. One of the most common questions to aging biology researchers is, “So, what do you take?”

Extending human life and health seems like a no‐brainer, and much of the public is fully supportive of this work. Yet there is a curious strain of antagonism toward the idea of longevity research, suggesting that this is somehow different from other medical research and is outright unethical. Arguments against aging research range from the existential (it will change the meaning of life) to the practical (we don't have enough resources for an even greater elderly population). If we address each of these arguments logically, however, we see that each implies untenable conclusions. Moreover, there are strong arguments for why human health and life have fundamental value and that incremental gains in them, from which individuals and societies have benefited often in the past, will continue to benefit us. From ethical and practical perspectives, we should support aging research and should, if we can, hang on, productively, past seventy‐five.

## Taking on the Arguments

***Death is “natural”; fighting death is “unnatural.”*** Critics of aging biology research often claim that death is “natural” and extending life and health is “unnatural.” The natural‐unnatural argument is that the aging process is inevitable and attempts to retard it are outside our basic biological design. This argument doesn't hold water.

To claim that attempts to change our current lifespan are unnatural is to ignore history. In 1860, the average lifespan in the United States, for men and women combined, was forty; in 1915, it was fifty‐four; now, it is seventy‐eight.^3^ What can it mean to say that the current average lifespan of seventy‐eight is natural, when this has been true for less than a century?

This argument also misrepresents the goal of aging research. Such research attempts, not to eliminate death, but to extend the time that one is able to live a healthy life. Many of the expected benefits of the research are in the extension of health span—healthy years—sometimes even without changing lifespan. Aging researchers say it is not inevitable that at fifty we start slowing down, at sixty‐five we retire, and at eighty we die. Why can't we be writing a novel at sixty‐five and tossing grandchildren over our heads at eighty?

Finally, if we reject scientific research that seeks to extend life as “unnatural,” then what other scientific research must we reject? The replacement of failing organs, generally regarded as a positive advance, is certainly not letting the body age as it would naturally. Is the administration of antibiotics or flu vaccination to the elderly unethical because it is unnatural? The logical—and untenable—conclusion of the “natural” versus “unnatural” argument is that we must eschew all medical intervention.

***Old people are a drain on society. Our retirement system can't handle paying for an additional twenty or forty years; our health system can't handle the additional load; and caring for older people is emotionally taxing***. Some worry that increasing the number of older people will be a drain on society, as it will financially tax our retirement, disability, and health care systems and will emotionally tax caretakers. The “drain on society” argument assumes that our imaginary 120‐year‐old spends almost half of his or her life suffering from various ailments and getting progressively sicker with each passing decade. But imagine instead a skilled surgeon who can operate for another thirty years or an award‐winning writer who pens five more books. Those who might not be well known but who enjoy putting in a good day's work would also have many more years to contribute and be active. Or those who want to retire from their original vocation might be able to spend time volunteering in needed areas. Few resource‐poor schools would turn down literacy volunteers; few legal services organizations would reject experienced lawyers as volunteers. Middle‐agers, instead of being stuck as part of the “sandwich generation,” caring for both children and parents, might instead receive childcare from healthy, doting grandparents. One could argue that, rather than taxing society, research that will help people live healthier longer will make it easier for parents to balance work and parenting; mothers, who still bear the lion's share of childcare responsibilities, could especially benefit from the career and educational opportunities that could open up as an indirect result of this research.

Indeed, respected economists like Andrew Scott,^4^ Dana Goldman,^5^ and Kevin Murphy and Robert Topel^6^ have looked at the economic impact of lifespan and health span extension and concluded that, rather than exacerbating negative economic effects of the coming so‐called gray wave of aging society, these improvements can be an answer to it. By prolonging the time people have for contributing to family life and society and by delaying and perhaps shortening the period of social and health care support needed, aging interventions could bring enormous economic gains in the trillions of dollars.

Granted, Social Security and retirement programs in the United States will need to be reconfigured if people live longer. That discussion is already ongoing, given that current programs were created when the average life expectancy was significantly lower than it is now. When Social Security was created in 1935, the retirement age of sixty‐five was higher than the average life expectancy. We can create a new system that provides a retirement safety net when people need it while acknowledging that not everyone will need it at the same time. The financial benefits from enabling a sizeable portion of our population to continue to contribute to society will vastly outweigh the additional costs in social and health care support.

***If people live longer, the world will overpopulate; we will use up our resources and harm the environment***. Another concern raised against aging biology research is that, if people live longer, necessary resources will be drained and the environment will suffer under the strains of overpopulation. Every day in the United States and around the world, people go hungry and thirsty and lack places to live. Nearly half of all energy produced by terrestrial plants now goes to human consumption,^7^ putting enormous strain on the natural environment, threatening species extinctions and ecosystem collapses. It can feel as if our resources are stretched beyond their limits and that adding more people, healthy or not, is a bad idea.

These are legitimate concerns, but the impact of the extension of lifespan and health span on population burden is not obviously negative. When the Gates Foundation embarked on a mission to reduce the burden of childhood mortality from malaria, critics complained that such efforts would drive overpopulation and lead to mortality from hunger and lack of other resources. While many of us would find this argument morally abhorrent, it also was factually wrong. As mortality from malaria decreased, birth rates decreased even further, such that total population growth actually declined, despite increased survival.^8^ This phenomenon, known as “demographic transition,” happens worldwide, and our longest‐lived societies, like Korea, Italy, and Japan, have birth rates so low that their populations are decreasing.^9^ The logical inference from this is that extension of lifespan is likely to reduce, not increase, the population burden on the world.

In addition, the longest‐lived societies take the most care to ensure sustainability.^10^ Perhaps because individuals in these societies have a better longitudinal view, they are better able to understand the long‐term benefits of caring for the planet. Whatever the reason, these longest‐lived societies have lower population growth rates (or even declines), and they also better manage the impact per capita on resource consumption, providing a double benefit of longer lifespan and health span on the world around them.

This is not to disregard the related issues we face today. Massive changes are required in how food is grown and distributed (an estimated 30 to 40 percent of the U.S. food supply is trashed), where and how we house people (reducing the percentage of single‐family houses on wide tracts of land), and how we care for our environment (with energy transition one of the most pressing concerns).^11^ But extensions to lifespan and health span will likely ameliorate, not exacerbate, environmental challenges overall, given that their effects are likely reductions to population growth and impact per capita.

***Spending money on aging research is a misallocation of health care resources***. We have limited health care dollars. Some argue that, given the number of people who suffer from a lack of health care resources, diverting money to people who don't suffer from a diagnosed disease but are experiencing only the natural progression associated with getting older is a cruel waste of these limited dollars. But who determines what is a “worthwhile” use of health care dollars? We do not demand that older people who suffer from cancer or heart disease refuse treatment to preserve health care dollars for a younger person. This is true even when someone with a low chance of survival pursues treatment. We have long placed the individual right to health atop the moral‐rights hierarchy and do not ask the patient to prove their value to society in order to qualify for treatment. Ageism, the implicit or explicit belief that an old life is somehow worth less, runs deep in our society. While in valuing the merit of an intervention it is important to consider how many years of benefit could result from it, this is exactly what interventions to extend lifespan and health span bring: more years of benefit.

Because aging affects all humans, aging biology research is one of the most egalitarian uses of health care resources. Unlocking the secrets of why our bodies stop behaving as they did when we were younger is the kind of basic research that has the potential to benefit an enormous number of people—all of us.

***The gap between the haves and the have‐nots will become even greater. Now, the wealthy will have more access to life itself***. Critics also worry that the gap between the haves and the have‐nots will increase if only wealthy people can take advantage of aging biology research. Here and in other countries, those with more money have better access to health care. They may have better access to cutting‐edge treatments if they have cancer, more disposable income to spend on medical devices and therapies, or even simply better coverage for routine procedures. And if we do not address this imbalance, those with more money may benefit more from lifespan and health span research. What's more, the perception that aging research is for the benefit of the rich alone is exacerbated by the funding and attention that some wealthy individuals have provided to the field.

The solution to an imbalance in health care distribution is not to cut off research or care for everyone but to work toward access for everyone. Policy‐makers should work to ensure that aging research benefits all income groups. Studies should include subjects from different economic as well as racial groups, and if, as hoped, the research yields actionable results, then efforts should be made to ensure that these are accessible to all.

Moreover, the interventions of aging research will likely be easier to democratize than other medical interventions are. The aphorism that “an ounce of prevention is worth a pound of cure” is instructive here. While it is difficult and expensive to intervene in late‐stage conditions like metastatic cancer and organ failure, early interventions to stay healthy are likely to be simpler to deliver and less expensive; hence, it should be easier and cheaper to ensure equity in access to them. For example, current aging research supports the idea that good sleep hygiene and a good network of social connections are two of the best things for people's health. These goods are free to cultivate and are available to most of us.

***Our current social structures depend upon our current life expectancy***. Much of society as we know it is constructed around the assumption that we live less than a century. If you knew you might be married to your current spouse for eighty years, would you still have married him or her? Would you still go to law school if it meant being a lawyer for sixty years? Some argue that our current marriage, educational, and employment choices are premised on the assumption that we may stay in them for only a relatively short period. But this criticism ignores the reality that social structures have already changed repeatedly.

As mentioned earlier, 150 years ago, life expectancy was shorter, and the average marriage did not last more than a few decades. But in 2020, 7.7 percent of currently married couples have celebrated their fiftieth anniversary.^12^ In response to changing life spans and gender norms over the past one hundred years, the average age of marriage for women has risen to 28.6 and for men to 30.5, and the divorce rate stands at about 41 percent for first marriages.^13^ Societal shifts in the average life expectancy may bring about more shifts in the average age of marriage or rates of divorce, but this is neither a new issue nor a potentiality that should weigh against conducting aging research. It is normal for cultural mores in family arrangements to shift over time for any number of reasons.

Estimates are that a current twenty‐something may have more than twelve jobs in their lifetime. A longer lifespan and health span might affect how we think about employment, but is that necessarily negative? As mentioned earlier, for those who choose to stay in their original profession, the opportunities for mentorship and shared experience could be enormous bonuses for new workers. Many older workers currently express frustration that their wisdom is not valued in the workforce. In a society where age is no longer falsely equated with decline, older workers will find their experience valued. And those workers who wish to change careers now have time to retrain. For parents who may have spent the first thirty years of adulthood trying to balance family and career, the chance to fully start or recharge a career at fifty or sixty is a gift, not a burden. Time is perhaps our most valuable resource, and while social structures will likely change if we have the ability to do more with longer time, insisting that they should not is backwards: our social structures are here to serve our collective needs.

***More older citizens will result in the stagnation of social and political systems. The old need to die to make space for the young to take control***. Some argue that more older citizens will result in the stagnation of social and political systems. In Japan, roughly 30 percent of the population is over sixty‐five.^14^ The sense that the elderly comprise a disproportionate share of the nation has given rise to the term “*rougai*,” which conveys the feeling that the older generation is holding power too long.^15^ In the United States, more than 50 percent of the Senate is over sixty‐five. A lack of term limits and a system that favors incumbents, it is argued, impedes turnover in the political system. The Nobel Laureate Max Planck noted in his autobiography that “a new scientific truth does not triumph by convincing its opponents and making them see the light, but rather because its opponents eventually die and a new generation grows up that is familiar with it.”^16^ How much longer could we be stuck with old dogmas—in science and broader aspects of society—if this mechanism of renewal is diminished because people are living longer?

Renewal of scientific thought and evolution of social and political norms are indeed laudable goals. Yet, as with other arguments against aging research, it seems a false assumption that extension of lifespan and health span necessarily compromises them. Few would argue that the pace of social and scientific change is slower now than in prior centuries, yet we are alive and healthier for many more years than people were in those periods. So, despite the concerns, we know empirically that longer lifespan does not inevitably slow the pace of change. Change and longevity can and do coexist.

However, if there are some downsides to longer life regarding the pace of change, it would seem a far better approach to institute mechanisms that ensure societal renewal alongside longer lifespan and health span, rather than to forgo the positive good of providing long, healthy life. For instance, to ensure political renewal, a simple mechanism exists in the form of term limits. These exist at the federal level for the presidency through the Twenty‐Second Amendment to the Constitution as well as at the state level in many jurisdictions, and they have at times been proposed for the Supreme Court and Congress. Similar approaches could be instituted, if necessary, in other realms of life. In the corporate world, the average tenure in the C‐suite of a Fortune 500 company is under five years, so little change would seem necessary there. In academia, where tenure provides for much longer terms, the tenure system will likely need adjustment.

***Life has meaning precisely because it is finite***. Perhaps the most stinging critique of longevity research is existential—that achieving its goal would make life less meaningful. Leon Kass has argued that “mortality makes life matter”—that is, life's finiteness helps give it meaning.^17^ He asserts that the knowledge that we will age and die gives meaning to our existence; by analogy, he observes that we have a fuller appreciation of the beauty of a flower because we know it will soon wither and die.

Kass's argument that finiteness imbues life with meaning is no challenge for lifespan and health span, as there is no prospect of immortality—merely additional valuable time. But even ignoring this point, Kass's argument falls flat. It implies a limited concept of what constitutes a meaningful life and sells the human imagination short. Yes, there is truth to the idea that we value what is limited; an original Renoir is more valuable than scores of excellent copies. But we do not value only that which is rare. A tulip in full bloom is beautiful, not because the petals will fade and drop off, but because we appreciate its bloom as an independent object of beauty. Most of us would be delighted if the tulip lasted an extra month.

For a number of philosophers, the meaningfulness of a life is measured by what one leaves behind. That may be children raised, canvases painted, money donated, tasks completed. In contrast to Kass's argument, more is sometimes better. While some people may continue on the career path they began, others may embark on new paths and find meaning in the fact that they can impact, not just one field, but two or more. Kass's view that the finiteness of an object adds to our sense of its value ignores the impact that more life can bring greater meaning to the individuals experiencing longer lives as well as those around them.

Research into extending lifespan and health span is sometimes seen as different from other kinds of health care. Some who are opposed to it raise the specter of an elderly population sucking out the marrow of the young, healthy minority and ruining the meaning of life. This boogeyman idea ignores the reality: human life and health are both intrinsically valuable and intrinsically interlinked. Research to improve and extend the period of life and health via aging biology is similar to other disease‐focused efforts in helping unlock this value. And the next wave may already be starting. For example, glucagon‐like peptide‐1 (GLP‐1) receptor agonists like the popular drug Ozempic are transforming health care but typically have not been categorized as part of aging biology. Researchers have been puzzled that these agents have demonstrated benefit across a wide range of diseases, exceeding what would be expected from just glucose control and weight loss.^18^ One hypothesis for this result is that GLP‐1 medications work in part by slowing the rate of aging itself and thus can prevent damage caused by many different diseases. This view is supported by the fact that these drugs slow the progress of all the so‐called hallmarks of aging.^19^ The point is we can begin to shift our mindset from thinking of aging biology as special and start to appreciate that understanding the mechanisms of aging can help in our understanding of disease, and vice versa.

What will remain different about the biology of aging, though, is that it matters to all of us; no one is exempt from aging. Longevity research has the power to help everyone, not just a subset with a specific disease, to live better and more fulfilled lives. So, Zeke, we do not need to choose whether to give more years to our lives or more life to our years—we can and should do both.

## Acknowledgment

Arthur L. Caplan acknowledges the support of the Hevolution Foundation for his work on ethics and geroscience.

## Disclosures

Caplan and Michael Ringel are both affiliated with the Hevolution Foundation. Michael Ringel, to whom Carolyn Ringel is married, is also affiliated with Life Biosciences and the American Federation for Aging Research.
